# In Situ Synthesis of Bimetallic Tungsten-Copper Nanoparticles via Reactive Radio-Frequency (RF) Thermal Plasma

**DOI:** 10.1186/s11671-018-2623-1

**Published:** 2018-07-24

**Authors:** Ji-Won Oh, Hyunwoong Na, Yong Soo Cho, Hanshin Choi

**Affiliations:** 1 Advanced Materials and Processing R&D Group, Korea Institute of Industrial Technology, Incheon, 21999 South Korea; 2 Materials Science and Engineering, Yonsei University, Seoul, 03722 South Korea

**Keywords:** Composites, Powders, Gas-phase reaction, Refractories, Radio-frequency (RF) thermal plasma

## Abstract

We synthesize, in situ, W-*x* wt% Cu (*x* = 5, 10, and 20 wt%) composite nanoparticles using inductively coupled radio-frequency (RF) thermal plasma. In the RF thermal plasma process, the W-*x* wt% Cu composite nanoparticles are synthesized by hydrogen reduction of tungsten trioxide (WO_3_) and cupric oxide (CuO). The synthesized W and Cu nanoparticles are effectively reduced to W and Cu, and the W-Cu nanoparticles are uniformly distributed bimetallic (or composite) nanoparticles.

## Background

W-Cu composites provide excellent performances in thermal/electrical management, offering high strength, high-temperature resistance, and other advantages [1–3]. The excellent physical properties of W-Cu composite present a high potential for use in automotive, aerospace, electric power, and electronic industries [4, 5]. Nevertheless, certain physical properties of W and Cu impede the fabrication of W-Cu composite materials.

The major issue in the fabrication process originates from the melting temperature of W and Cu. W has very high melting temperature (*T*_m_) of 3683 K with a low thermal expansion coefficient; Cu melts at 1353 K but offers high thermal/electrical conductivity. The huge difference between *T*_m_ (W) and *T*_m_ (Cu) makes hard to fabricate W-Cu composite materials. In addition, W-Cu has no mutual solubility and high contact angle, so W-Cu-based composites, in general, have difficulty achieving full densification by liquid-phase sintering [6]. On the other hand, their different physical properties give a wide range to select the material’s properties by changing the ratio between the W and Cu contents. For example, W-*x* wt% Cu with *x* < 20 wt% is used for electrical/thermal management, such as in electric circuits and wiring, and for components in ceramic-based electronic devices [7]. W-*x* wt% Cu with *x* < 80 wt% is used for high-power electricity contact materials and heat sinks for high-density integrated circuits [8–10].

Recently, W-Cu composite nanoparticles have been investigated in order to reduce the size of applied products. Widely used processes for W-Cu nanoparticles are mechanical milling [2, 5, 11], thermochemical methods [12], and chemical synthesis [7]. These methods, however, are still limited to reducing the particle size in a spherical shape with homogeneous distribution of W-Cu composite nanoparticles. Another barrier of W-Cu nanoparticles is the low densification that occurs during the sintering process [13]. In other W-based composite alloys, such as in the W-Ni binary system, W has a small amount of solubility in Ni [14], so the additional densification is induced by Ostwald ripening during the sintering process [15, 16]. By contrast, the W-Cu binary system cannot undergo a further sintering mechanism to improve the degree of densification because of immiscibility. Kim et al. recently suggested that Cu nanoparticle-coated W micro-powder improves densification during the sintering process by liquid-phase sintering [9]. Due to its lower melting point, the Cu component melts and infiltrates into the green body pores by capillary force which, in turn, enhances the densification. This previous study, therefore, suggests that the barriers to synthesize W-Cu composite nanoparticles can be overcome by structural design of W-Cu composite nanoparticles.

Based on the previous report, inductively coupled radio-frequency (RF) thermal plasma was used to synthesize the W-*x* wt% Cu composite nanoparticles in order to improve the microstructural uniformity and densification in the sintered W-Cu. As mentioned above, the dispersion of Cu in the process of liquid-phase sintering of the W-Cu composite closely affects densification [9]. Therefore, it is expected that Cu can improve the sintering property of liquid phase by preparing nanoparticles of core-shell structure through heterogeneous condensation reaction on W surface. In our study, we synthesized W-*x* wt% Cu (*x* = 5, 10, and 20 wt%) and investigated the synthesized W-20 wt% Cu nanoparticles from a macroscopic to microscopic scale. The microstructural investigation showed that the nanoparticles are formed by nucleation from supersaturated gaseous species and spherically grown by heterogeneous condensation and/or collision-coalescence process [17].

## Methods

Feedstock micro-powders were prepared by blending tungsten trioxide (WO_3_, > 99.99% purity; LTS Inc., New York, USA) and cupric oxide (CuO, > 99.99% purity; LTS Inc., New York, USA) micro-powders for the 5, 10, and 20 wt% Cu in the weight fraction. The blended micro-powders (feedstock powders) were dried at 423 K for 1 h before feeding. WO_3_ and CuO micro-powders were selected as precursors to synthesize the W and Cu nanoparticles due to the low melting temperatures. WO_3_ and CuO have much lower boiling points (WO_3_, 1973 K; CuO, 2273 K) than those of W (5828 K) and Cu (2835 K); this means that the fed micro-powders are more easily vaporized through the RF thermal plasma process (30 kW induction plasma system; Tekna, Quebec, Canada) compared to pure W and Cu metal powders. In addition, the oxidized micro-powder prevents oxidization when the material is exposed to air.

In the next process, hydrogen gas was used to reduce the vaporized feedstock. W and Cu nanoparticles were then obtained by using a quenching gas, which cools down a hot gas and accelerates nucleation kinetics. Hydrogen gas was passed through argon sheath gas, and nitrogen gas was injected to quench the vaporized gas and to accelerate nucleation kinetics. Based on the above processes, the experimental conditions were determined to satisfy the full vaporization and reduction of WO_3_ and CuO micro-powders (Table 1).

**Table 1:** Process parameters for RF plasma synthesis

Variables	Samples	Invariables						
		Power (kW)	Pressure (psi)	Central gas: Ar (slpm)	Carrier gas: Ar (slpm)	Sheath gas 1: Ar (slpm)	Sheath gas 2: H_2_ (slpm)	Feed-rate (g min^−1^)
Weight fraction $\frac{\mathrm{CuO}}{\mathrm{CuO}+{\mathrm{WO}}_3}$	0.05	28	14.7	15	5	60	10	5
	0.10							
	0.20							

*slpm* standard liters per minute

## Results

**Fig. 1:**
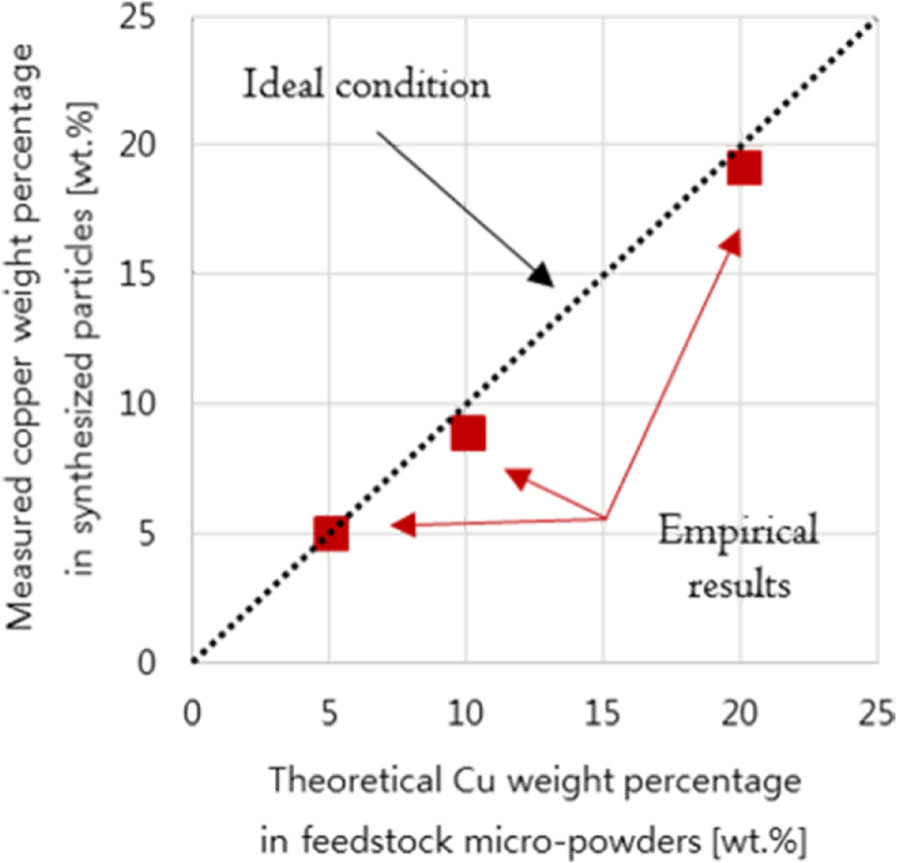
Overall chemical composition of the as-synthesized nanoparticles

We first measured the overall chemical composition of the synthesized W-*x* wt% Cu (*x* = 5, 10, and 20 wt%) nanoparticles using scanning electron microscopy (SEM)-EDS (Quanta 200F, FEI, Oregon, USA). In the blended feedstock, the WO_3_ and CuO micro-powders were respectively prepared to have W-5 wt% Cu, W-10 wt% Cu, and W-20 wt% Cu in the synthesized W-Cu nanoparticles. The nominal compositions were obtained from each blended feedstock and then compared to the synthesized W-Cu nanoparticles. As shown in Fig. 1, the chemical compositions of the blended feedstocks well agree with those of synthesized W-*x* wt% Cu nanoparticles.

**Fig. 2:**
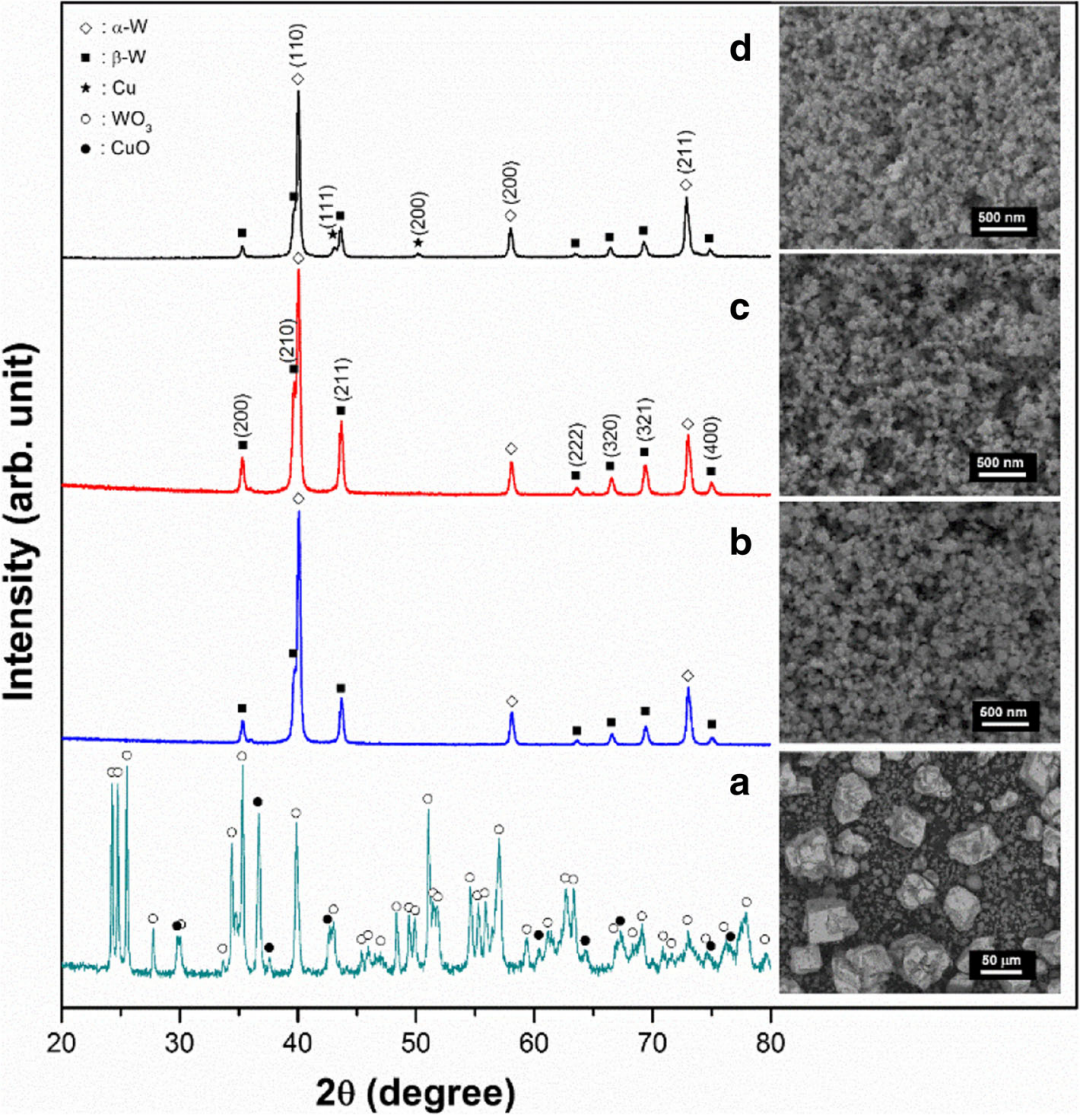
XRD profiles corresponding inset SEM images. **a** WO_3_ and CuO micro-powder blended feedstock. **b** As-synthesized W-5 wt% Cu nanoparticle. **c** As-synthesized W-10 wt% Cu nanoparticle. **d** As-synthesized W-20 wt% Cu nanoparticle

Figure 2 shows the X-ray diffraction (XRD; D8 DISCOVER, Bruker Inc., Darmstadt, Germany) profiles recorded from synthesized W and Cu nanoparticles with the representative morphologies recorded using SEM (inset). As can be seen in Fig. 2a, the feedstock micro-powder only consists of WO_3_ and CuO with irregular shapes (inset of Fig. 2a). By using the blended feedstock (WO_3_ and CuO), the W and Cu nanoparticles were then synthesized for W-5 wt% Cu, W-10 wt% Cu, and W-20 wt% Cu. As shown in Fig. 2b–d, the synthesized W-(5, 10, 20) wt% Cu composite powders are indexed with α-W (bcc, $\operatorname{Im}\overline{3}m$), W_3_O (or β-W) (A15 structure, Pm3n) [18], and Cu (fcc, $\mathrm{Fm}\overline{3}m$). Thus, the used oxide powders (WO_3_, CuO) are mostly reduced by the hydrogen gas while the oxidized W_3_O (β-W) is observed in all W-*x* wt% Cu nanoparticles. Nevertheless, the metastable β-W is transformed into an α-W stable phase from room temperature to ~ 900 K by the removal of oxygen atoms from the β-matrix. It is, therefore, evident that the β-W can be fully reduced during the sintering process [19].

**Fig. 3:**
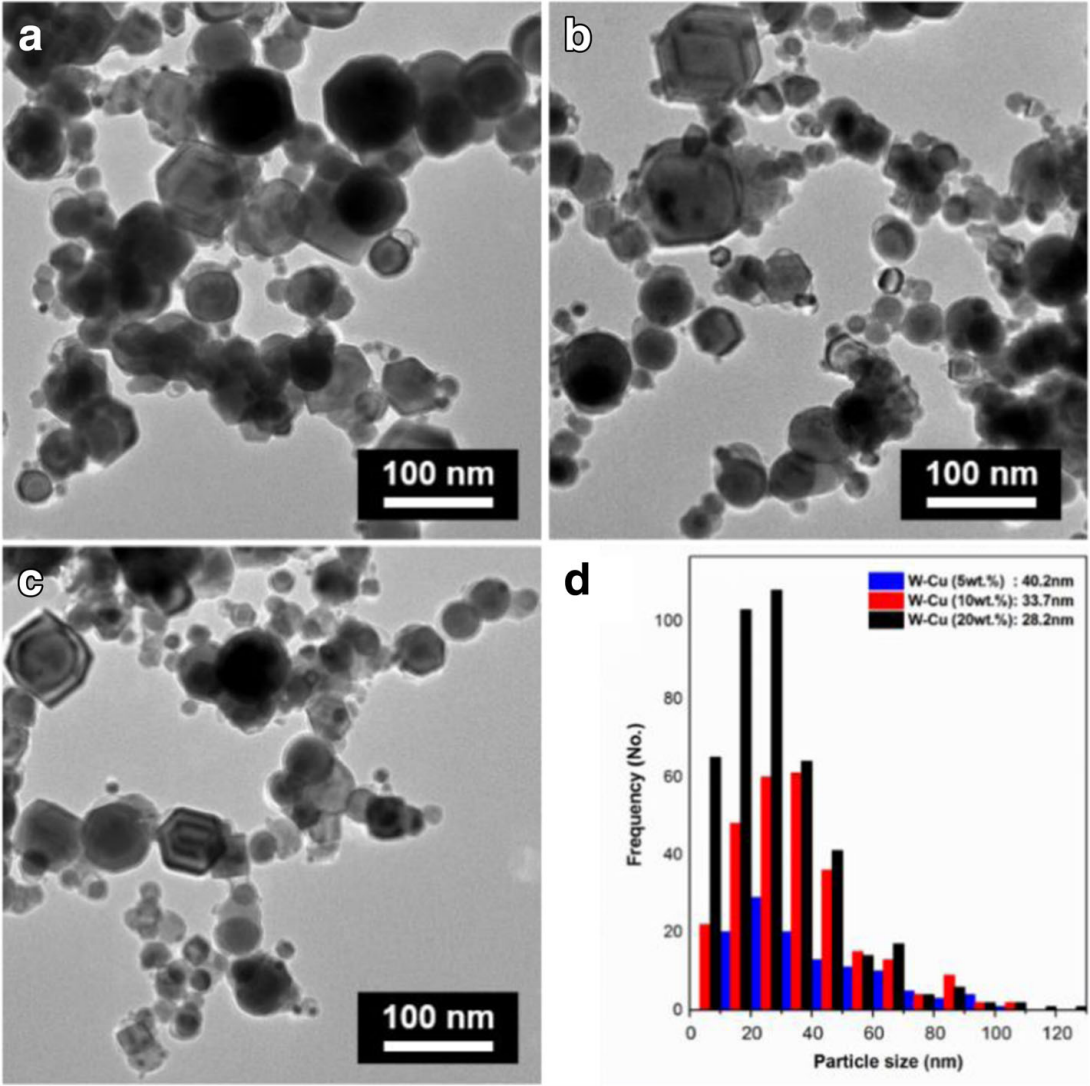
TEM images of as-synthesized **a** W-5 wt% Cu nanoparticles, **b** W-10 wt% Cu nanoparticles, **c** W-20 wt% Cu nanoparticles, and **d** particle-sized distribution of each particle, respectively

In the microstructural aspect, the cuboid and spherical W-Cu nanoparticles are well observed in the transmission electron microscopy (TEM) images (Fig. 3) with average particle sizes of 28.2 nm (W-5 wt% Cu), 33.7 nm (W-10 wt% Cu), and 40.2 nm (W-5 wt% Cu), as represented in Fig. 3d. The particle size distribution of the prepared particles was measured from the TEM image by the diameter of the sphere of equivalent cross-sectional area.

**Fig. 4:**
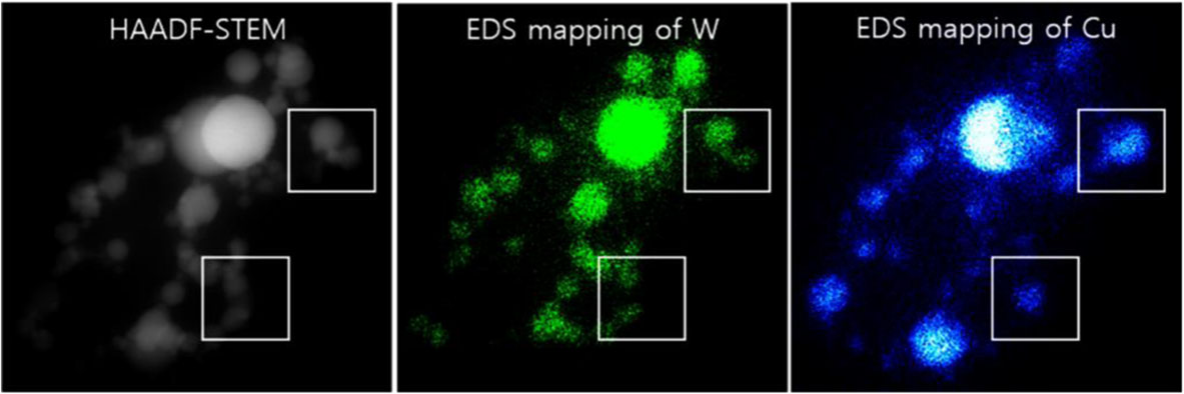
Elemental mapping of the W-20 wt% Cu nanoparticle using EDS on STEM analysis

The distribution of W and Cu nanoparticles is investigated at a microscopic scale by using SEM with energy-dispersive X-ray spectroscopy (EDX). The overall chemical composition was recorded from several regions in W-20 wt% Cu, which is almost identical to the chemical composition as shown in Fig. 1. Figure 4 shows a typical high-angle annular dark-field (HAADF) scanning transmission electron microscopy (STEM) image of W-20 wt% Cu nanoparticles with the elemental mapping result. The elemental maps for W and Cu show that the W and Cu nanoparticles are individually synthesized. In addition, the synthesized W and Cu nanoparticles are uniformly dispersed as the bimetallic nanoparticles.

**Fig. 5:**
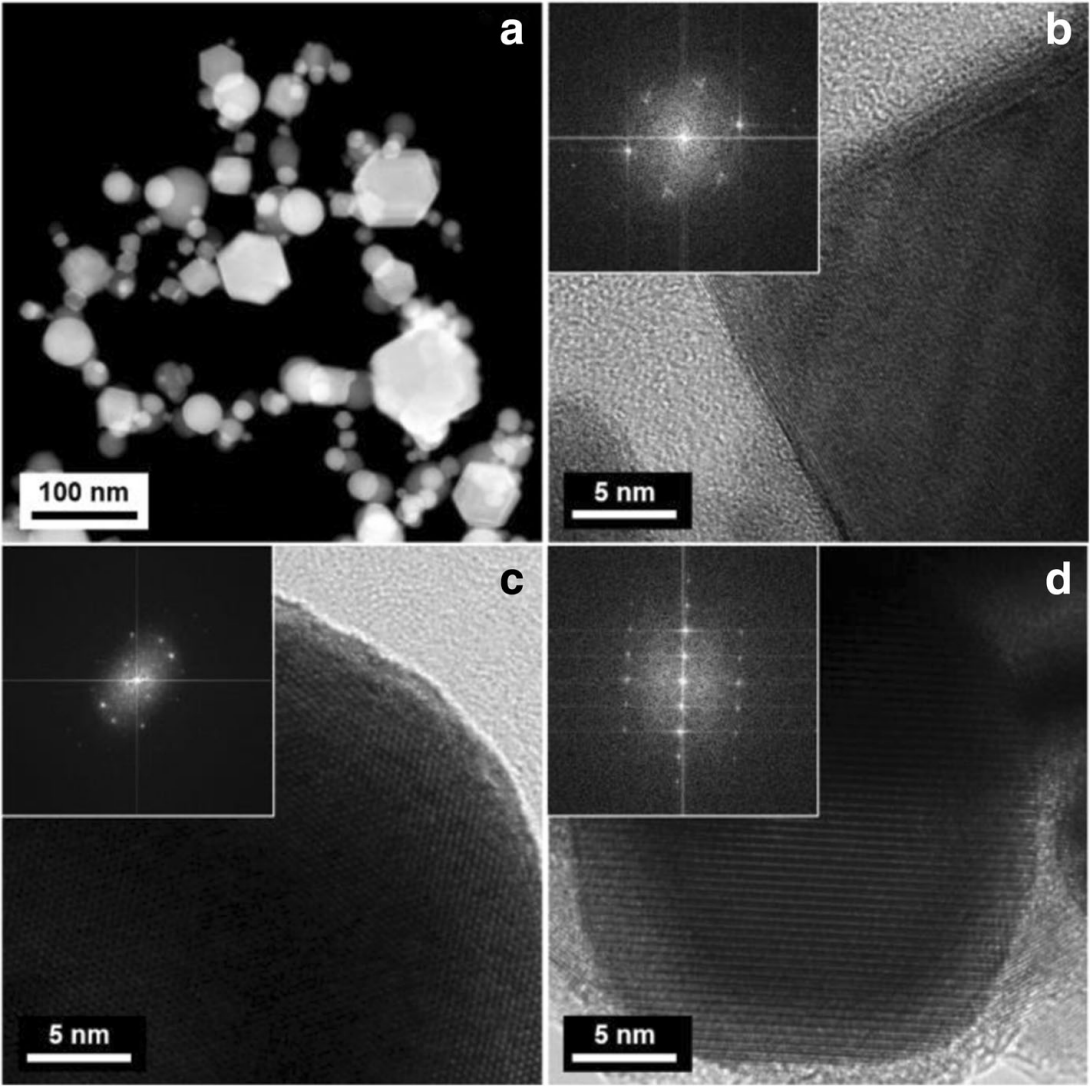
**a** A typical HAADF STEM image of the as-synthesized W-20 wt% Cu nanoparticles. **b** High-resolution (HR) TEM image of a representative α-W and its corresponding Fourier-filtered transformed (FFT) diffraction pattern for α-W. **c** HRTEM image of a representative β-W and its corresponding FFT pattern for β-W. **d** HRTEM image of a representative Cu and its corresponding FFT pattern for Cu

Based on the chemical analysis, the relation between morphology and structure was investigated. Figure 5 shows the typical bright-field (BF) image recorded from the W-20 wt% Cu nanoparticles. Structural investigation was carried out for three phases (α-W, β-W, and Cu) found in the XRD profiles. Figure 5a shows a representative morphology of α-W phase observed in the synthesized W-20 wt% Cu nanoparticles. Based on the indexing result of the power spectrum (inset), α-W mostly exists in the form of a cuboid, as shown in Fig. 5b. On the other hand, β-W and Cu phases are, in general, spherical, as shown in Fig. 5c, d.

## Discussion

In this study, we used WO_3_ and CuO as the feedstock powder due to their lower melting temperatures compared to pure W and Cu. The blended feedstock was then vaporized and reduced by hydrogen. W and Cu nanoparticles were individually synthesized from WO_3_ micro-powder and CuO micro-powder, since their vaporization and condensation procedures can be different. Nucleation of nanoparticles is dependent on the thermophysical properties, vapor pressures, and cooling rate of gaseous species. Stably nucleated nanoparticles are further grown by heterogeneous condensation of gaseous species in the remaining vapor and/or collision-coalescence process of in-flight nanoparticles. By considering the melting temperature of W and Cu, the W nanoparticles were first nucleated at higher gas temperatures and the nucleation of Cu nanoparticles followed from the remaining Cu-rich vapor during cooling. The heterogeneous condensation and/or collision-coalescence reactions between W and Cu nanoparticles then resulted in composite nanoparticles. Because of the poor wettability of Cu, island growth of Cu on the surface of the W nanoparticle was expected during the heterogeneous condensation of Cu. When the W and Cu nanoparticles were individually generated and collided, coagulation to single particle was difficult to obtain because of their mutual insolubility. Consequently, the W-Cu nanoparticles were in situ synthesized in the form of bimetallic nanoparticles, as shown in Fig. 4.

**Fig. 6:**
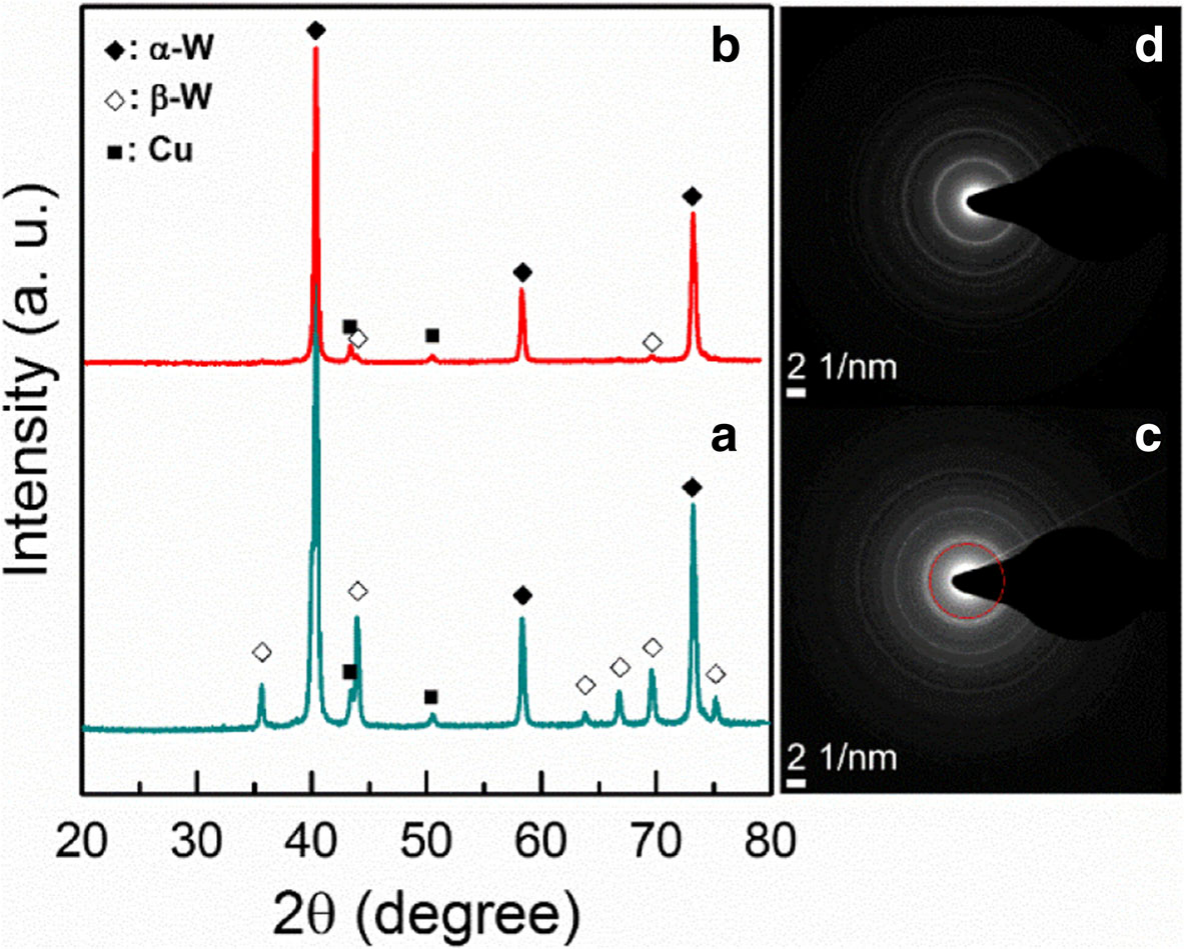
**a**, **b** XRD profiles and **c**, **d** SADP images for as-synthesized and heat-treated at 1073 K W-20 wt% Cu nanoparticles

Partially unreduced β-W was observed in the synthesized W-Cu nanoparticles. It has been reported that the metastable β-W is transformed into an α-W stable phase at high temperatures [19–22]. To further reduce the observed β-W, we heat treated the W-20 wt% Cu nanoparticles at 1073 K in a hydrogen environment. As shown in the XRD profiles of Fig. 6b, the fraction of β-W phase drastically decreased at the temperature of 1073 K. We also investigated the existence of the β-W phase at a microscopic scale. Figure 6c, d shows the selected area diffraction patterns (SADPs) recorded from the as-synthesized and heat-treated W-20 wt% Cu nanoparticles. The SADP of the specimen shows the diffracted spots of (200) β-W, while the specimen heat treated at 1073 K had no spots of β-W. From the above results, therefore, it has been established that synthesized W and Cu nanoparticles can be fully reduced during a sintering process.

## Conclusions

We in situ synthesized W-*x* wt% Cu (*x* = 5, 15, and 20 wt%) using a RF thermal plasma process. The spherical- and cuboid-shaped W-*x* wt% Cu composite nanoparticles are obtained by the reduction of WO_3_- and CuO-blended feedstock micro-powders and the post-heat treatment. From the elemental composition analyses, the ratios of W and Cu are approximately consistent with the blended feedstocks. This is because both feedstock micro-powders are fully vaporized and effectively reduced via the RF thermal plasma process. In addition, the different nucleation paths of W and Cu result in a uniformly synthesized W-*x* wt% Cu, bimetallic nanoparticles, despite difficulties in the fabrication of W-Cu composites due to the immiscibility of the metals. From the above results, we believe that this study provides a technique for any immiscible elements to be synthesized into bimetallic nanopowders using the RF thermal plasma process.
